# In This Issue

**Published:** 1997

## ALCOHOL’S EFFECTS ON LIVER FUNCTION

Long-term heavy alcohol use is the most prevalent single cause of illness and death from liver disease in the United States. Dr. Jacquelyn J. Maher discusses possible mechanisms of alcohol-induced liver injury and explores current and potential treatments. Serious liver injury occurs in people who have been drinking heavily for many years. The risk for such injury may be increased by genetic variation, diet, gender, and hepatitis C infection. Research has led to innovative treatments for alcoholic liver disease, including the use of corticosteroids, antioxidants, antibiotics, and certain polyunsaturated fats. Increased understanding of the disease process may provide additional new approaches to treating the problem. (pp. 5–12)

## ALCOHOL-RELATED PANCREATIC DAMAGE

Pancreatitis is a potentially fatal inflammation of the pancreas that is often associated with long-term alcohol consumption. Drs. Minoti V. Apte, Jeremy S. Wilson, and Mark A. Korsten discuss the development and treatment of both the acute and the chronic forms of this disease. Research has revealed several possible mechanisms of alcohol-induced pancreatic damage, such as the release of digestive enzymes within the pancreas, which leads to destruction of tissue. Treatment of pancreatitis is difficult and largely directed at ameliorating symptoms. Additional research on the cause and progression of alcoholic pancreatitis may lead to more effective treatment methods or a potential cure. (pp. 13–20)

## ALCOHOL AND THE CARDIOVASCULAR SYSTEM

Alcohol consumption has been reported to have both positive and negative effects on the cardiovascular system. In this article, Dr. Sam Zakhari presents the contrasting effects of alcohol on the heart. He begins by summarizing current research findings on light-to-moderate drinking and the mechanisms that may “protect” against coronary artery disease. Dr. Zakhari then discusses possible mechanisms that contribute to alcohol’s negative effects on the cardiovascular system, including heart muscle disorders, heart rhythm irregularities, high blood pressure, and strokes. (pp. 21–29)

## ALCOHOL’S CONTRIBUTION TO COMPROMISED IMMUNITY

The immune system is an intricate network of many types of blood cells and proteins that protect the body against infections from bacteria, viruses, and other invaders. These defense mechanisms can be severely impaired in alcoholics, leaving them susceptible to a variety of infections, including pneumonia and tuberculosis, and compromising their ability to fight infections once they occur. Dr. Gyongyi Szabo provides an overview of the immune system and its functions and reviews alcohol’s effects on different immune-system components. (pp. 30–41)

## THE HEMATOLOGICAL COMPLICATIONS OF ALCOHOLISM

Alcohol adversely affects the production and function of all types of blood cells, placing heavy drinkers and alcoholics at increased risk for disease. Most important, alcohol-induced decreases in the numbers and activities of white blood cells increase the drinker’s risk of serious infections, explains Dr. Harold S. Ballard. Moreover, alcohol-related abnormalities in the production and structure of red blood cells can result in anemia, causing symptoms that range from fatigue to reduced mental capacity. Finally, alcohol interferes with the production and function of the platelets, which play a crucial role in blood clotting. (pp. 42–52)

## THE ENDOCRINE SYSTEM: ALCOHOL ALTERS CRITICAL HORMONAL BALANCE

The effects of alcohol on the hormonal (i.e., endocrine) system have far-reaching consequences. Alcohol-related hormone imbalances can lead to immune dysfunction, cardiovascular abnormalities, bone disease, and reproductive problems in men and women of all ages. Drs. Nicholas and Mary Ann Emanuele review the effects of both acute and chronic alcohol exposure on hormonal physiology, addressing research performed in humans and in animals. The authors also describe innovative research that may uncover ways of using hormonal mechanisms to aid in the treatment of alcoholism. (pp. 53–64)

## IMPAIRMENTS OF BRAIN AND BEHAVIOR

Prolonged heavy alcohol consumption can cause abnormal mental functioning and changes in behavior. Alcohol can affect the brain both directly and through damage to other organs that affect brain function. Drs. Marlene Oscar-Berman, Barbara Shagrin, Denise L. Evert, and Charles Epstein review alcohol’s effects on the nervous system, summarizing the biochemical effects of alcohol at all levels, from molecular interactions to the brain as a whole. These effects include changes in emotions and personality, as well as impaired perception, learning, and memory. Improved understanding of alcohol’s actions on nerve cells may assist in the development of treatments for the effects of alcohol on the nervous system. (pp. 65–75)

## ALCOHOL’S ROLE IN GASTROINTESTINAL TRACT DISORDERS

The gastrointestinal (GI) tract is the first organ system to come into contact with alcohol after the consumption of alcoholic beverages. As a result, drinkers can experience numerous alcohol-induced disorders of the mouth, esophagus, stomach, and intestines. Drs. Christiane Bode and J. Christian Bode describe some of the mechanisms underlying these effects. For example, alcohol damages the cell layers lining the GI tract and impairs the movement of the muscles surrounding the GI organs. Moreover, alcohol-induced alterations in the intestinal walls allow passage of toxins into the blood, damaging the liver and other organs. (pp. 76–83)

## ALCOHOL’S IMPACT ON KIDNEY FUNCTION

The kidneys have a dual role in maintaining health: They excrete the body’s waste products and keep the volume and composition of body fluids in exact balance. Both chronic and acute alcohol consumption can interfere with these vital functions, however. Dr. Murray Epstein describes alcohol’s effects on kidney structure, function, and hormonal regulation. Although some alcohol-induced effects are modest, serious kidney dysfunction can occur if excessive drinking progresses to liver disease. According to Dr. Epstein, three of the most prominent kidney disturbances that occur in the presence of established liver disease are sodium retention, an impaired ability to excrete excess fluid, and a form of acute kidney failure known as hepatorenal syndrome. (pp. 84–92)

